# δ-Aminolevulinic Acid Dehydratase Polymorphism and Risk of Brain Tumors in Adults

**DOI:** 10.1289/ehp.7986

**Published:** 2005-05-10

**Authors:** Preetha Rajaraman, Brian S. Schwartz, Nathaniel Rothman, Meredith Yeager, Howard A. Fine, William R. Shapiro, Robert G. Selker, Peter M. Black, Peter D. Inskip

**Affiliations:** 1 Division of Cancer Epidemiology and Genetics, National Cancer Institute, National Institutes of Health, Department of Health and Human Services, Bethesda, Maryland, USA; 2 Johns Hopkins Bloomberg School of Public Health, Baltimore, Maryland, USA; 3 Core Genotyping Facility, and; 4 Neuro-oncology Branch, National Cancer Institute, National Institutes of Health, Department of Health and Human Services, Bethesda, Maryland, USA; 5 Barrow Neurological Institute, St. Joseph’s Hospital and Medical Center, Phoenix, Arizona, USA; 6 Western Pennsylvania Hospital, Pittsburgh, Pennsylvania, USA; 7 Brigham and Women’s Hospital, Boston, Massachusetts, USA

**Keywords:** ALAD, brain, case–control, meningioma, polymorphism, tumor

## Abstract

The enzyme δ -aminolevulinic acid dehydratase (ALAD), which catalyzes the second step of heme synthesis, can be inhibited by several chemicals, including lead, a potential risk factor for brain tumors, particularly meningioma. In this study we examined whether the *ALAD G177C* polymorphism in the gene coding for ALAD is associated with risk of intracranial tumors of the brain and nervous system. We use data from a case–control study with 782 incident brain tumor cases and 799 controls frequency matched on hospital, age, sex, race/ethnicity, and residential proximity to the hospital. Blood samples were drawn and DNA subsequently sent for genotyping for 73% of subjects. *ALAD* genotype was determined for 94% of these samples (355 glioma, 151 meningioma, 67 acoustic neuroma, and 505 controls). Having one or more copy of the *ALAD2* allele was associated with increased risk for meningioma [odds ratio (OR) = 1.6; 95% confidence interval (CI), 1.0–2.6], with the association appearing stronger in males (OR = 3.5; 95% CI, 1.3–9.2) than in females (OR = 1.2; 95% CI, 0.7–2.2). No increased risk associated with the *ALAD2* variant was observed for glioma or acoustic neuroma. These findings suggest that the *ALAD2* allele may increase genetic susceptibility to meningioma.

The *ALAD* gene codes for the enzyme δ -aminolevulinic acid dehydratase (ALAD), which catalyzes the second step of heme synthesis involving the condensation of two molecules of aminolevulinic acid (ALA) to form porphobilinogen. The most commonly studied polymorphism in the gene, *ALAD G177C* (dbSNP ID: rs1800435) contains a G-to-C transversion at position 177 of the coding region, resulting in the substitution of asparagine for lysine. *ALAD G177C* has two codominant alleles: *ALAD1* and *ALAD2* (Battistuzzi et al. 1981), with an *ALAD2* allele prevalence of approximately 10% (range, 6–20%) in Caucasian populations, 3–11% in Asian populations, and 3% in African-American populations (Kelada et al. 2001).

Although ALAD can be inhibited by a variety of chemicals, including lead, trichloroethylene, bromobenzene, and styrene (Fujita et al. 2002), polymorphic differences in enzyme binding or chemical uptake have been examined most extensively for lead. On average, individuals with the *ALAD2* allele have higher blood lead levels than do *ALAD1* homozygotes, probably due to tighter binding of lead by the ALAD2 enzyme (Alexander et al. 1998; Bergdahl et al. 1997; Fleming et al. 1998; Hsieh et al. 2000; Shen et al. 2001; Wetmur et al. 1991; Ziemsen et al. 1986). Previous studies in animals and humans indicate that exposure to lead may increase the risk of brain tumors [International Agency for Research on Cancer (IARC) 1987; Silbergeld 2003; Steenland and Boffetta 2000], particularly meningioma (Cocco et al. 1999; Hu et al. 1999; Navas-Acien et al. 2002).

Given that the *ALAD G177C* polymorphism affects the toxicokinetics of lead in the body, and that exposure to lead may increase the risk of adult brain tumors, we postulated a possible association of *ALAD G177C* genotype and risk of intracranial tumors of the brain and nervous system (hereafter referred to as brain tumors). Analyses were conducted using data from a hospital-based case–control study of brain tumors conducted by the National Cancer Institute (NCI) between 1994 and 1998.

## Materials and Methods

### Study population.

Subjects for the brain tumor study were enrolled from 1994 through 1998 from three hospitals specializing in brain tumor treatment, located in Phoenix, Arizona; Boston, Massachusetts; and Pittsburgh, Pennsylvania. The study protocol was approved by the institutional review board of each participating institution. Study methods have been described in detail elsewhere (Inskip et al. 2001).

Eligible cases for the parent study were ≥ 18 years of age with a first intracranial glioma, meningioma, or acoustic neuroma diagnosed during or within the 8 weeks preceding hospitalization. Ninety-two percent of eligible brain tumor patients agreed to participate in the study. All diagnosed cases of glioma and meningioma were confirmed by microscopy, as were 96% of acoustic neuroma cases. A total of 489 subjects with glioma, 197 subjects with meningioma, and 96 subjects with acoustic neuroma were enrolled.

Controls were patients admitted to the same hospitals as cases for a variety of non-neoplastic conditions, with the most common being injuries (25%), circulatory system disorders (22%), musculoskeletal disorders (22%), and digestive disorders (12%). More than 90% of patients were interviewed within 1 year of symptom onset. Control subjects were frequency matched in a 1:1 ratio to all brain tumor cases based on hospital, age, sex, race/ethnicity, and proximity of residence to the hospital; 799 controls (86% of all contacted controls) were enrolled.

Informed written consent was obtained from all cases and controls. Blood samples were collected and sent for genotyping for 73% of all subjects: 382 subjects with glioma (78%), 158 subjects with meningioma (80%), 71 subjects with acoustic neuroma (74%), and 540 control subjects (68%). The main obstacle to obtaining blood samples was subject refusal, with nonparticipation in the blood draw being higher for control subjects than for case subjects.

Shortly after hospitalization, a trained research nurse administered a structured in-person interview for each subject. Information on known or possible risk factors for brain tumors (including a detailed occupational history) was collected for all subjects.

### Processing of blood samples.

DNA was extracted from the peripheral white blood cells (buffy coat or granulocytes) of blood samples using a phenol-chloroform method described by Daly et al. (1996). *ALAD* genotyping was conducted by the NCI’s Core Genotyping Facility using a medium-throughput TaqMan assay (Applied Biosystems, Foster City, CA).

Reactions for the assay were done in a 384 (96 × 4)-well plate format. Lyophilized sample DNA (10 ng) was used for a 5 μL TaqMan reaction. Four Coriell DNA controls (Coriell Cell Repositories, Camden, NJ) for each genotype as well as no-template controls (NTCs) were put on the plate along with the samples; 2.5 μL of the 2X Universal Master Mix (Applied Biosystems), 200 nM of each primer, and 900 nM of each probe was used in the reaction. Probe 1 (TGTGAAGCGGCTGG), specific to the *ALAD1* allele, contained the FAM dye reporter. Probe 2 (TGTGAACCGGCTGG) was specific to the *ALAD2* allele and contained the VIC dye reporter. The primers used were primers F (TGCCTTCCTTCAACCCCTCTA) and R (CAAGGGCCTCAGCATCTCTT). Step 1 of the assay-specific thermocycling process involved 2 min of UNG (uracil-DNA glycosylase) activation using AmpErase UNG (Applied Biosystems) at 50°C. This was followed by 10 min of enzyme activation at 95°C (step 2), 0.30 sec of template denaturation at 92°C if using 3′MGB quencher, or at 95°C if using 3′TAMRA quencher (step 3), and 1 min of assay-specific annealing at 60°C (step 4). Steps 3 and 4 were repeated 49 times, after which the reaction was held at 4°C. The plate was then read on the ABI 7900HT sequence detection system (Applied Biosystems), and the results of the allelic discrimination were graphed as a scatter plot of allele 1 reaction versus allele 2 reaction (SDS Software; Applied Biosystems), with each well of the 384-well plate represented as a spot on the graph. Four distinct clusters on the allelic plot represented the NTCs and three possible genotypes, *ALAD1–1*, *ALAD2–2,* and *ALAD1–2*, respectively. Calls were determined manually by a technician using SDS software.

Quality control specimens for the study included multiple samples from three individuals who were not study subjects (QC-A, *n* = 34; QC-B, *n* = 20; QC-C, *n* = 15) and 76 duplicates from study subjects. These specimens were submitted for genotyping in a masked fashion and were collected and processed in a manner identical to that for study samples. Ninety-eight percent agreement was achieved between the three non-study replicates. The concordance rate for study duplicates (study samples compared with masked relabels) was 87% for glioma, 100% for meningioma, 85% for acoustic neuroma, and 89% for controls.

Using a conservative call strategy that labeled a call as missing if the genotype was unclear, *ALAD* genotyping was successfully conducted for 94% of samples (93% of gliomas, 96% of meningiomas, 94% of acoustic neuromas, and 94% of controls). Missing values (noncalls) were generally equally likely to be from case or control samples.

### Statistical analysis.

We assessed statistically significant departure from Hardy-Weinberg equilibrium for controls using the chi-square test. Unconditional logistic regression was used to estimate odds ratios (ORs) and calculate 95% confidence intervals (CIs) for the effect of the variant *ALAD2* allele, adjusting for study matching factors. We entered adjustment variables as indicator variables in the following categories: age in years (18–29, 30–39, 40–49, 50–59, 60–69, 70–79, 80–99); race/ethnicity (non-Hispanic white, Hispanic, African-American, other); sex (male, female); hospital (Phoenix, Boston, Pittsburgh); and residential proximity to the hospital in miles (0–4, 5–14, 15–29, 30–49, ≥ 50). Because the small number of *ALAD2–2* homozygotes precluded accurate estimation of risk for *ALAD1–2* heterozygotes and *ALAD2–2* homozygotes separately, these categories were combined for analysis. *ALAD1–1* homozygotes were the reference group. In order to test for the influence of control group composition on the results, the models were run excluding each major category of control discharge diagnosis, one at a time.

## Results

The distribution of demographic characteristics for genotyped subjects (Table 1) was comparable with the distribution for all study subjects (Inskip et al. 2001). Most demographic characteristics were distributed similarly for brain tumor cases and controls. Relative to controls, subjects with glioma were proportionately more often male, whereas subjects with meningioma and acoustic neuroma were more often female. Differences between individual tumor group and control group distributions were mostly due to the matching of controls for all tumors combined rather than for specific tumor subgroups.

Study participants in the youngest and oldest age brackets were less likely to have given a blood sample, as were control subjects who lived closest to the hospital. Brain tumor cases that were male, of “other” race/ethnicity, or from the Phoenix study site were also less likely to have given a blood sample (results not shown).

No significant departure from Hardy-Weinberg equilibrium was detected for controls (*p* = 0.3). Table 2 summarizes the association between ALAD G177C genotype and risk of each brain tumor type. The odds of meningioma were significantly higher for individuals possessing any ALAD2 allele compared with ALAD1–1 homozygotes (OR = 1.6; 95% CI, 1.0–2.6). This risk did not differ markedly when different control subgroups (musculoskeletal disorders, circulatory disorders, digestive disorders, or trauma) were excluded. Although the observed association between ALAD2 and meningioma appeared stronger in males (OR = 3.5; 95% CI, 1.3–9.2) than in females (OR = 1.2; 95% CI, 0.7–2.2), the sample size for the sex-specific estimates was small, with only 10 male and 25 female meningioma cases possessing the variant allele. No increased risk associated with the ALAD2 variant was observed for glioma or acoustic neuroma.

## Discussion

We found that the ALAD2 allele of the G177C polymorphism was associated with increased risk of meningioma, especially in males. Confirmation of our findings will require replication in other studies with a larger number of meningioma cases. If risk of meningioma is truly increased in individuals with the ALAD2 allele, the question arises as to whether the effect depends upon exogenous chemical exposures that act on the heme synthesis pathway or is independent of such exposures. A direct effect of the ALAD2 polymorphism might be indicated if the ALAD2 allele has lower enzyme activity than the ALAD1 allele, given that the precursor ALA is thought to be neurotoxic and genotoxic (Silbergeld 2003). However, ALAD enzyme activity does not appear significantly different for the two alleles (Battistuzzi et al. 1981). Alternatively, it is possible that the increased risk of meningioma in ALAD2 individuals arises in the presence of chemicals that influence the heme synthesis pathway. Several chemicals have been shown to inhibit ALAD enzyme activity, including lead, trichloroethylene, bromobenzene, and styrene (Fujita et al. 2002). Polymorphic differences in enzyme binding or chemical uptake have been examined most extensively for lead, and individuals with the ALAD2 allele are generally reported to have higher blood lead levels than are individuals with the ALAD1 allele (Alexander et al. 1998; Bergdahl et al. 1997; Fleming et al. 1998; Hsieh et al. 2000; Shen et al. 2001; Wetmur et al. 1991; Ziemsen et al. 1986).

The observation that the relationship between ALAD2 and risk of meningioma was stronger in men than in women could be due to biologic differences or differential exposure to a chemical agent modified by ALAD genotype. Given the small number of male meningioma cases with the variant allele, we also cannot rule out the possibility that the observed effect modification is due to chance.

The specific question of ALAD genotype and brain tumor risk has not been addressed previously in the literature. In a previously published analysis of this same data set, we found elevated risk of meningioma in individuals who had worked in military occupations or as autobody painters, designers and decorators, industrial production supervisors, teachers, or managers (Rajaraman et al. 2004). Aside from teachers and managers, all of these occupations have potential exposure to lead, suggesting that lead might be implicated in meningioma risk. Our observation of increased risk with the ALAD2 variant for meningioma, but not for glioma or acoustic neuroma, parallels the observation that reports of increased risk of brain tumor with lead have been more consistent for meningioma (Cocco et al. 1999; Hu et al. 1999; Navas-Acien et al. 2002) than for glioma or for all brain tumors combined. However, the role of lead in the observed association between ALAD genotype and meningioma can be meaningfully addressed only when data on both ALAD genotype and individual lead exposure are available.

In a study with hospital controls, study results can be biased if the exposure under study is associated with conditions enrolled in the control series (Miettinen 1985). To assess for such a bias, we conducted a sensitivity analysis by excluding one major control subgroup at a time from the analysis. Systematically excluding control subgroups did not change observed ORs appreciably and resulted, if anything, in slightly stronger evidence of an association between ALAD2 and meningioma when circulatory or digestive disorders were excluded. If use of hospital controls introduced a bias in the observed OR, therefore, the likely direction of the bias was toward the null.

The relatively low concordance rate for study duplicates is another potential concern. However, the concordance for duplicates from meningioma cases was 100%, so our observed association for meningioma was probably not affected by genotyping concerns. Moreover, if we assume that 10% nondifferential misclassification did occur, this would have biased our findings toward the null, making our observed association a conservative estimate.

We chose to study the ALAD polymorphism based on a priori biologic and functional considerations and not by screening a large number of associations. Nonetheless, the possibility that our findings are due to chance cannot be ruled out. These findings should be viewed as hypothesis generating and need to be confirmed by replication in other studies.

Although our study had limited power for evaluating risk with respect to subtypes of tumor, it remains one of the largest case–control studies of brain tumors to date. Aside from a small percentage of brain tumors that can be explained by familial syndromes or exposure to ionizing radiation, very little is known about the etiology of brain tumors (Preston-Martin and Mack 1996; Wrensch et al. 2002). In order to clarify the role of lead (or other chemicals) in the observed relationship between *ALAD* genotype and risk of meningioma, it will be important to conduct a detailed exposure assessment and evaluate the joint effect of exposure and *ALAD* genotype in this, or another, study population.

## Figures and Tables

**Table 1:** Demographic characteristics for individuals with glioma, meningioma, and acoustic neuroma and frequency-matched controls^a^ for genotyped individuals: NCI adult brain tumor study, 1994–1998.

Characteristic	Glioma cases (*n* = 355)	Meningioma cases (*n* = 151)	Acoustic neuroma cases (*n* = 67)	Controls (*n* = 505)
Sex				
Male	192 (54.1)	32 (21.2)	23 (34.3)	234 (46.3)
Female	163 (45.9)	119 (78.8)	44 (65.7)	271 (53.7)
Race/ethnicity				
White, non-Hispanic	323 (91.0)	123 (81.5)	61 (91.0)	450 (89.1)
Hispanic	19 (5.4)	12 (8.0)	5 (7.5)	36 (7.1)
Black	7 (2.0)	9 (6.0)	0 (0.0)	11 (2.2)
Other	6 (1.7)	7 (4.6)	1 (1.5)	8 (1.6)
Mean age (years)	51.3	55.0	51.6	49.4
Hospital site				
Phoenix, AZ	162 (45.6)	75 (49.7)	49 (73.1)	258 (51.1)
Boston, MA	126 (35.5)	62 (41.1)	18 (26.8)	164 (32.5)
Pittsburgh, PA	67 (18.9)	14 (9.3)	0 (0.0)	83 (16.4)

Values are no. (%) except where indicated.

a Controls were matched to the total case group including glioma, meningioma, and acoustic neuroma.

**Table 2:** Association of ALAD2 polymorphism (ALAD1–2 or ALAD2–2 ) with risk of glioma, meningioma, and acoustic neuroma: NCI adult brain tumor study, 1994–1998.^a^

	Glioma (*n* = 355)		Meningioma (*n* = 151)		Acoustic neuroma (*n* = 67)		Control (*n* = 505)
	No. (%)^b^	OR (95% CI)	No. (%)	OR (95% CI)	No. (%)	OR (95% CI)	No. (%)
ALAD1–1	301 (84.8)	1.0	116 (76.8)	1.0	57 (85.1)	1.0	420 (83.2)
ALAD1–2	53 (14.9)	0.9 (0.6–1.3)^c^	32 (21.2)	1.6 (1.0–2.6)^c^	10 (14.9)	0.9 (0.4–1.9)^c^	79 (15.6)
ALAD2–2	1 (0.3)		3 (2.0)		0		6 (1.2)

a Models adjusted for matching factors (hospital, sex, race/ethnicity, age, residential proximity to hospital); results are only reported if number of exposed cases is ≥ 5.

b Percentages based on genotyped samples.

c Estimates are for having ≥ 1 copy of the ALAD2 allele.
